# Cross-Domain Feature Similarity Guided Blind Image Quality Assessment

**DOI:** 10.3389/fnins.2021.767977

**Published:** 2022-01-14

**Authors:** Chenxi Feng, Long Ye, Qin Zhang

**Affiliations:** ^1^Key Laboratory of Media Audio and Video, Ministry of Education, Communication University of China, Beijing, China; ^2^State Key Laboratory of Media Convergence and Communication, Communication University of China, Beijing, China

**Keywords:** cross-domain feature similarity, image quality assessment, deep learning, transfer learning, human visual system

## Abstract

This work proposes an end-to-end cross-domain feature similarity guided deep neural network for perceptual quality assessment. Our proposed blind image quality assessment approach is based on the observation that features similarity across different domains (e.g., Semantic Recognition and Quality Prediction) is well correlated with the subjective quality annotations. Such phenomenon is validated by thoroughly analyze the intrinsic interaction between an object recognition task and a quality prediction task in terms of characteristics of the human visual system. Based on the observation, we designed an explicable and self-contained cross-domain feature similarity guided BIQA framework. Experimental results on both authentical and synthetic image quality databases demonstrate the superiority of our approach, as compared to the state-of-the-art models.

## 1. Introduction

Objective image quality assessment (IQA) aims to enable computer programs to predict the perceptual quality of images in a manner that is consistent with human observers, which has become a fundamental aspect of modern multimedia systems (Zhai and Min, 2020). Based on how much information the computer program could access from the pristine (or reference) image, objective IQA could be categorized into full-reference IQA (FR-IQA) (Wang et al., 2003, 2004; Sheikh and Bovik, 2006; Larson and Chandler, 2010a; Li et al., 2011; Zhang et al., 2011, 2014; Liu et al., 2012; Chang et al., 2013; Xue et al., 2013), reduced-reference IQA (RR-IQA) (Wang and Simoncelli, 2005; Wang and Bovik, 2011; Rehman and Wang, 2012), and no-reference (or blind) IQA (NR-IQA/BIQA) (Kim and Lee, 2016; Liu et al., 2017; Ma et al., 2017a; Lin and Wang, 2018; Pan et al., 2018; Talebi and Milanfar, 2018; Sun et al., 2021). The absence of reference information in most real-world multimedia systems calls for BIQA methods, which are more applicable but also more difficult.

Deep neural network (DNN) has significantly facilitated various image processing tasks (Fang et al., 2017; Park et al., 2018; Casser et al., 2019; Ghosal et al., 2019) in recent years due to its powerful capacity in feature abstraction and representation. It is also worth noting that the success of deep-learning techniques is derived from large amounts of training data, which is often leveraged to adjust the parameters in the DNN architecture to guarantee that both the accuracy and generalization ability are satisfying. Unfortunately, image quality assessment is typically a small-sample problem since the annotation of the ground-truth quality labels calls for time-consuming subjective image quality experiments (Zhang et al., 2018a). Inadequate quality annotations severely restrict the performance of DNN-based BIQA models in terms of both accuracy and generalization ability.

In order to address the problem caused by limited subjective labels, data augmentation is firstly employed to increase the training labels (e.g., Kang et al. (2014)) proposed to split the image with quality labels into multiple patches. and each of the patches is assigned with a quality score which is the same with the whole image. However, some distortion types are inhomogeneous, i.e., the perceptual quality of local patches might differ from the overall quality of the whole image. Therefore, transfer learning has gained more attention to relieve the small-sample problem (Li et al., 2016). Specifically, the BIQA framework is comprised of two stages: which are pre-training and fine-tuning. In the pre-training stage, the parameters in the DNN architecture are trained by other image processing tasks such as object recognition, whilst in the fine-tuning stage, images with subjective labels are employed as training samples. Such a transfer-learning scheme is feasible since the low-level feature extraction procedure across different image processing tasks are shared (Tan et al., 2018).

More recently, various sources of external knowledge are incorporated to learn a better feature representation for the BIQA issue. For example, hallucinated reference (Lin and Wang, 2018) is generated via a generative network and employed to guide the quality-aware feature extraction. The distortion identification is incorporated as the auxiliary sub-task in MEON model (Ma et al., 2017b), by which the distortion type information is transparent to the primary quality prediction task for better quality prediction. Visual saliency is employed in Yang et al. (2019) to weight the quality-aware features more reasonably. Semantic information is also employed for better understanding of the intrinsic mechanism of quality prediction, e.g., multi-layer semantic features are extracted and aggregated through several statistical structures in Casser et al. (2019). An effective hyper network is employed in Su et al. (2020) to generate customized weights from the semantic feature for quality prediction, i.e., the quality perception rule differs as the image content changes.

**Figure 1:**
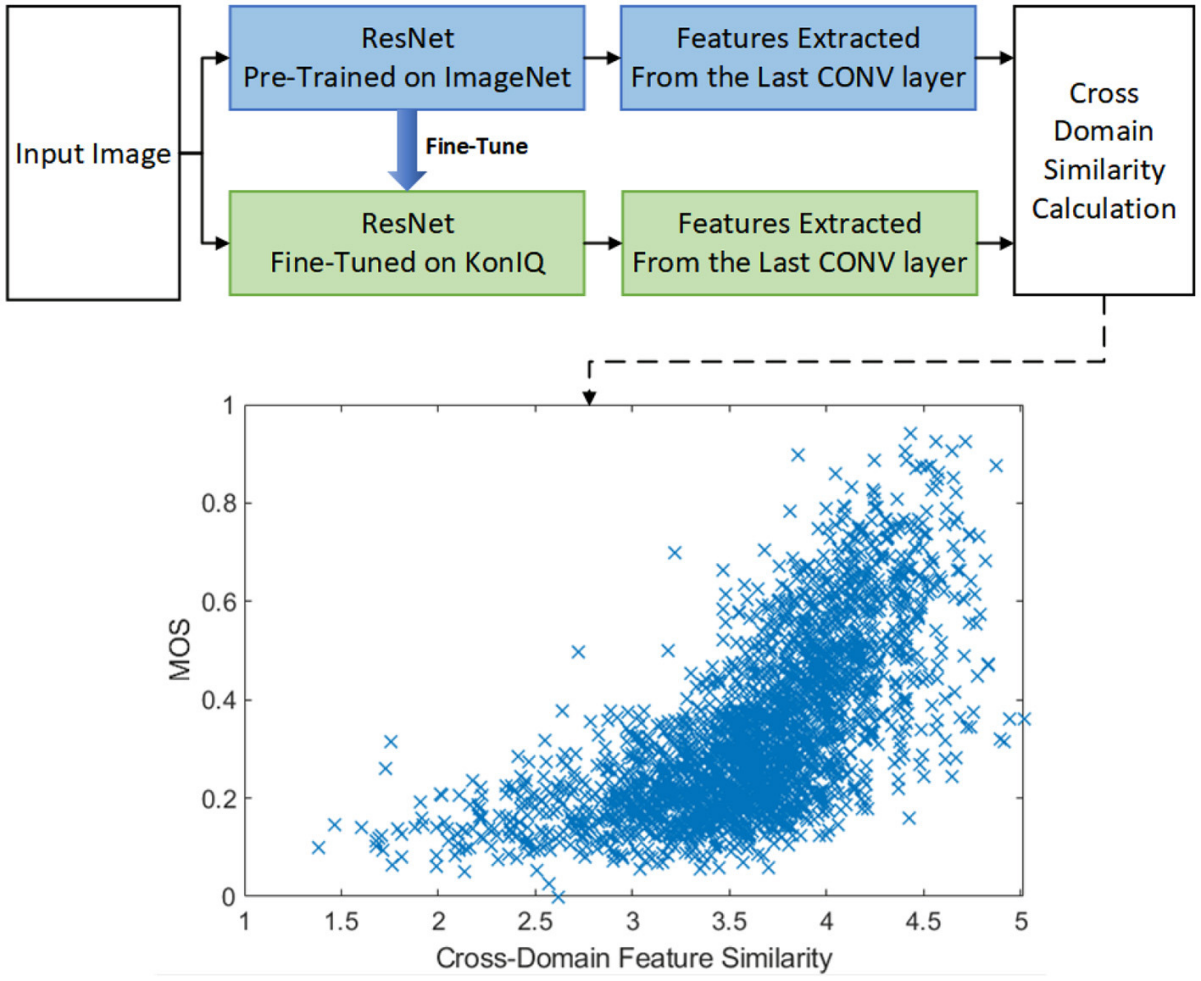
The overall framework of our proposed CDFS guided BIQA approach. As shown in the lower part, the cross-domain feature similarity is highly correlated with the perceptual quality. The ‘cross-domain similarity calculation’ is obtained by: (1) Extractinged the features from the last convolutional layer of pre-trained ResNet (denoted as *R*_*s*_) and fine-tuned ResNet (denoted as *R*_*q*_); (2) Calculatinge the similarity matrix *W* according to Equation 1; (3) Obtaining the eigen values of *W* by $\vec{v}=eig\left(W\right)$; (4) The similarity *Sim* is calculated by $Sim=\frac{1}{std\left(v\right)}$, in which *std*(·) denotes the standard deviation operator.

Unlike other studies, this paper employs the cross-domain feature similarity as an extra restraint for better quality-aware feature representation. Specifically, the transfer-learning based BIQA approach is pre-trained in one domain (say, object recognition in the semantic domain) and is fine-tuned in the perceptual quality domain with similar DNN architectures, we have observed that the cross-domain (Semantic vs. Quality) feature similarity would, in turn, contribute to the quality prediction task (as shown in Figure 1).

**Figure 2:**
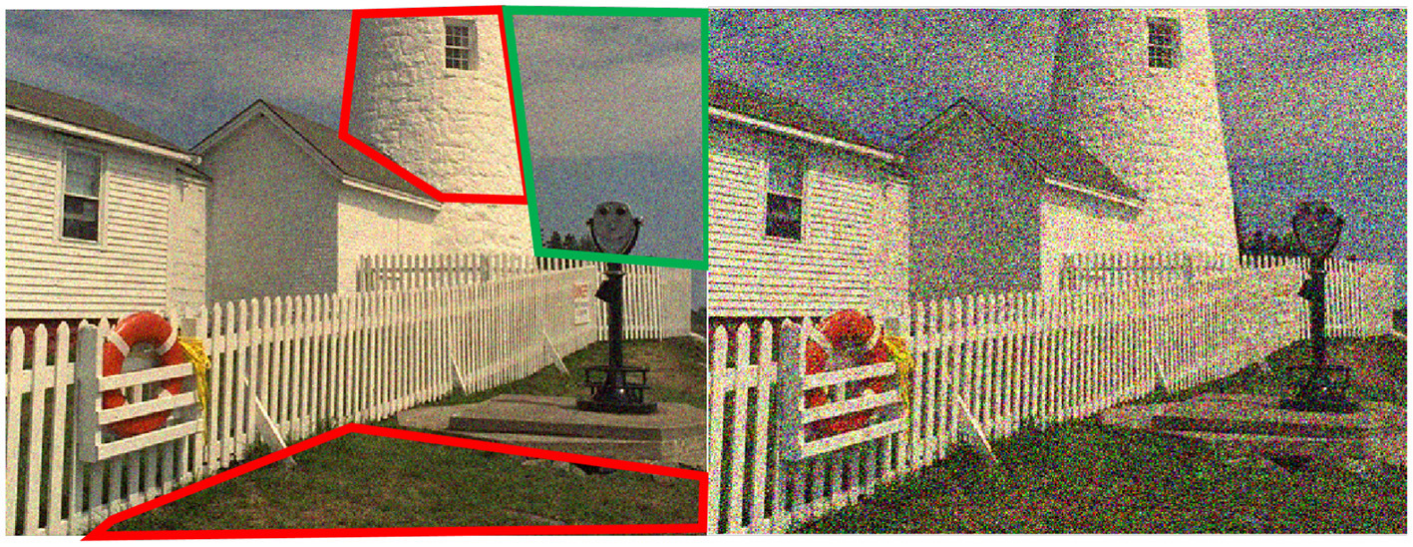
Illustration of different strategies that the human visual system would take to assess the perceptual quality when viewing images with different amounts of degradation. Specifically, when judging the quality of a distorted image containing near-threshold distortions (Left), one tends to rely primarily on visual detection of any visible local differences, e.g., the distortions in red boxed are slighter than that in the green box even though the noise intensity is the same. On the other hand, when judging the quality of a distorted image with clearly visible distortions (Right), one would rely much less on visual detection and much more on overall image appearance e.g., the distortions in each image area are roughly the same.

By thoroughly analyzing the intrinsic interaction between object recognition task and quality prediction task, we think the phenomenon represented in Figure 1 is sensible. As shown in Figure 2, previous works (Larson and Chandler, 2010b) have revealed that human observers would take different strategies to assess the perceptual quality when viewing images with different amounts of degradation: when judging the quality of a distorted image containing near-threshold distortions, one tends to rely primarily on visual detection of any visible local differences, in such a scenario, semantic information is instructive for quality perception since distortion in the semantic-sensitive area would contribute more in the quality decision and vice versa. On the other hand, when judging the quality of a distorted image with clearly visible distortions, one would rely much less on visual detection and much more on the overall image appearance, in such a scenario, the quality decision procedure is much more independent with semantic information.

**Figure 3:**
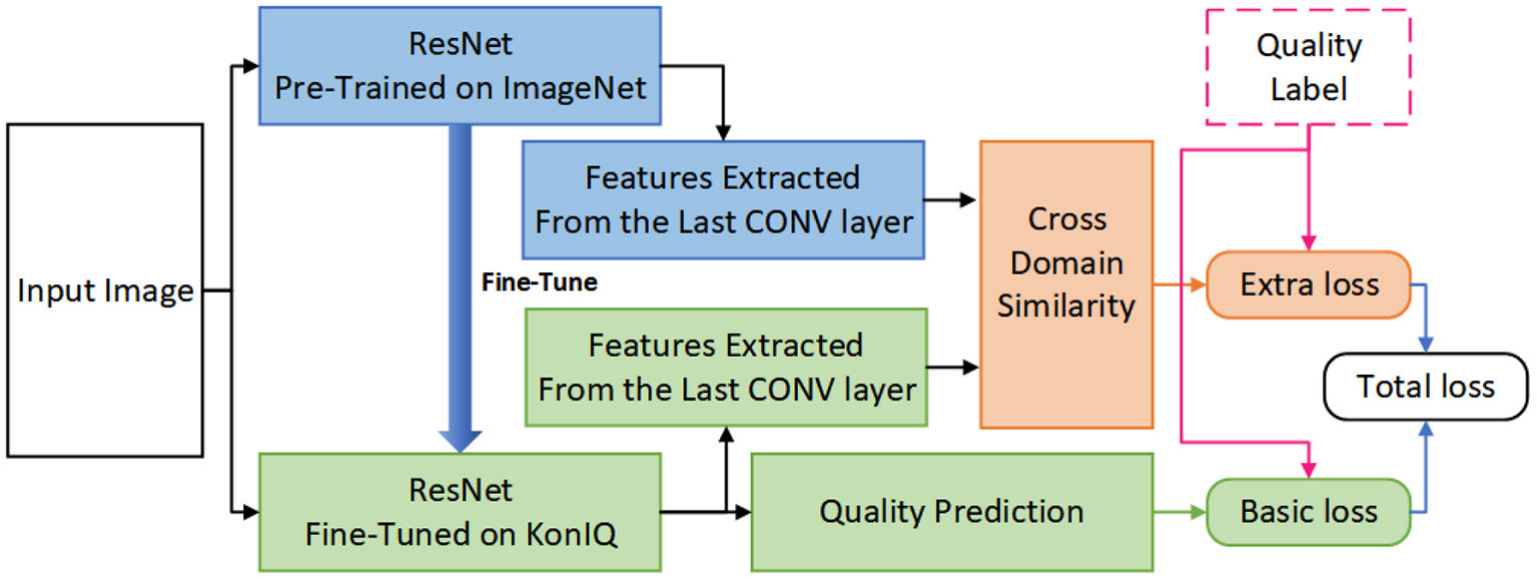
The overall pipeline of our proposed CDFS-based IQA approach.

Considering the effectiveness of cross-domain feature similarity (CDFS), this work leverages CDFS as an extra restraint to improve the prediction accuracy of BIQA models. As shown in Figure 3, the parameters in our CDFSNet are updated according to both the basic loss and the extra loss, which would restrain the network yielding quality predictions as similar as the ground-truth label whilst maintaining that the CDFS also correlates well with the perceptual quality, in such a manner that, the accuracy of the DNN architecture would get improved according to the experimental results presented in section 3.

Compared to the aforementioned works, the superiority of the cross-domain feature similarity guided BIQA framework is embodied in the following aspects:

1. The proposed cross-domain feature similarity is self-contained for transfer-learning based BIQA models since the transfer-learning procedure itself is comprised of the training in two different domains (i.e., object recognition and quality prediction). Therefore, no extra annotation procedure (such as distortion identification in Ma et al., 2017b and visual saliency in Yang et al., 2019) is needed.
2. The proposed cross-domain feature similarity is more explicable since it is derived from the intrinsic characteristic of interactions between semantic recognition and quality perception.
3. In addition to general-purpose IQA, the performance of our proposed CDFS guided BIQA framework is also evaluated on other specific scenarios such as screen content (Xiongkuo et al., 2021) and dehazing oriented (Min et al., 2018b, 2019) IQA. The experimental results indicate that CDFS guided BIQA has significant potential toward diverse types of BIQA tasks (Min et al., 2020a,b).

The rest part of the paper is organized as follows: Section 2 illustrates the details of our CDFS-based BIQA framework and section 3 shows the experimental results; Section 4 is the conclusion.

## 2. Materials and Methods

### 2.1. Problem Formulation

Let *x* denote the input image, conventional DNN based BIQA works usually leverage an pre-trained DNN architecture *f*(·;θ) (with learnable parameters θ) to predict the perceptual quality of *x* via $\hat{q}=f\left(x;\theta \right)$, where $\hat{q}$ denotes the prediction of perceptual quality *q*.

Our work advocates employing the cross-domain feature similarity to supervise the update of parameters in a quality prediction network. Specifically, let *f*(·;θ_*Smtc*_) denotes the DNN with fixed and pre-trained parameters oriented toward semantic recognition, and *f*(·;θ_*Qlty*_) denotes the DNN with learnable parameters oriented toward quality prediction. It should be noticed that *f*(·;θ_*Smtc*_) and *f*(·;θ_*Qlty*_) share the same architectures whilst having own different parameters. This work attempts to further improve the quality prediction accuracy by analyzing the similarity between the features extracted for different tasks, i.e., features extracted for semantic recognition *ft*_*s*_ = *f*(*x*; θ_*Smtc*_), and features extracted for quality regression *ft*_*q*_ = *f*(*x*; θ_*Qlty*_).

Given three-dimensional features *ft*_*s*_ and *ft*_*q*_ with size [*C, H, W*], where *C*, *H*, *W* denotes the channel size, height, and width of the features, respectively, *ft*_*s*_ and *ft*_*q*_ are firstly reshaped into *R*_*q*_ and *R*_*s*_ with size [*C, H* × *W*]. The similarity *Sim* between *R*_*q*_ and *R*_*s*_ is obtained via the following steps.

***Step 1***, employ linear regression to express *R*_*q*_ via *R*_*s*_, i.e., *R*_*q*_ = *W* × *R*_*s*_ + *e*, where *W* denotes the weighting matrix and *e* denotes the prediction error of linear regression. Therefore, *W* could be obtained by


(1) $$\begin{array}{r} W={\left(R_{s}^{T}\times R_{s}\right)}^{-1}\times R_{s}^{T}\times R_{q} \end{array}$$

***Step 2***, a learnable DNN architecture *g*(·;γ) is employed to yield the similarity between *ft*_*s*_ and *ft*_*q*_ given *W*, i.e., *Sim* = *g*(*W*; γ)

### 2.2. Network Design

**Figure 4:**
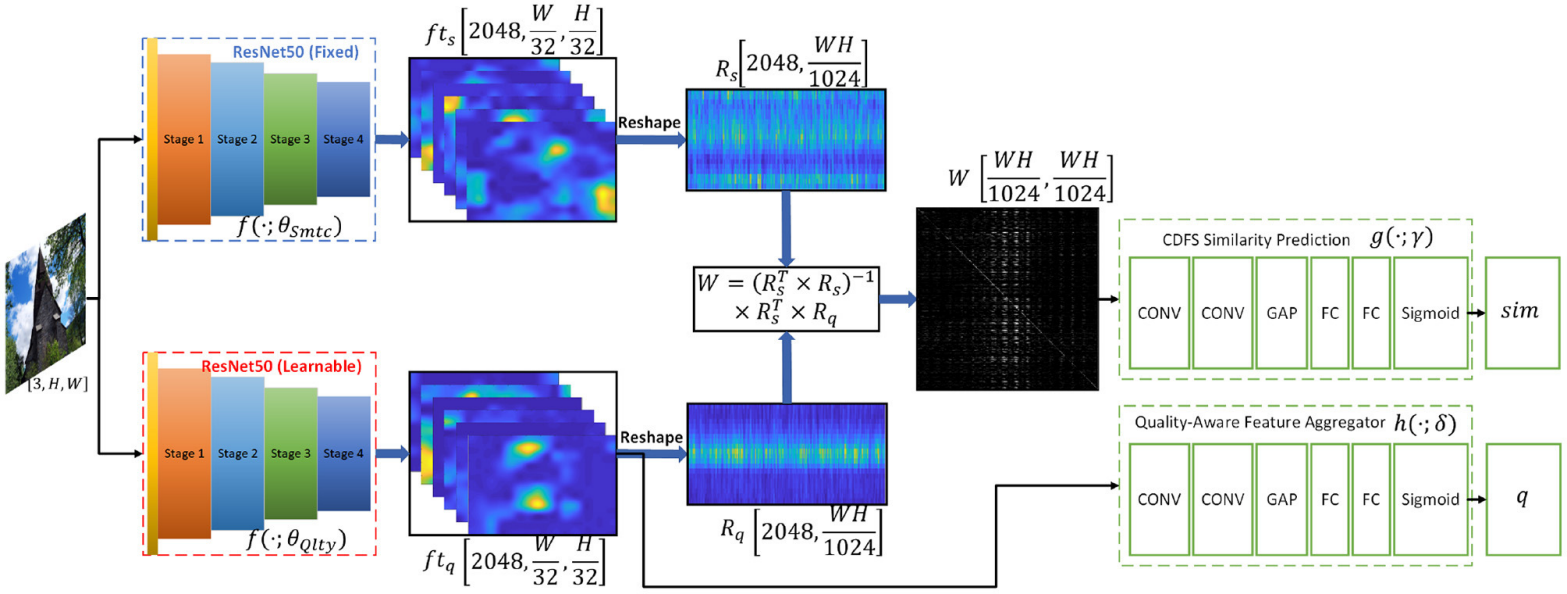
The detailed architecture of our proposed Cross-Domain Feature Similarity Guided Network. The “CONV” denotes convolutional layers followed by batch normalization and ReLU layer, the “FC” denotes the fully-connected layer, and the ‘GAP’ denotes the global average pooling layer.

The architecture of our proposed network is shown in Figure 4, which mainly consists of a semantically oriented feature extractor, perceptual-quality oriented feature extractor, and cross-domain feature similarity predictor. More details are described as follows.

#### 2.2.1. Semantic Oriented Feature Extractor

The DNN pre-trained in large-scale object recognition datasets (e.g., ImageNet Deng et al., 2009) are leveraged as the semantic oriented feature extractor.

Specifically, this work employs the activations of the last convolutional layers in ResNet50 to represent the semantic-aware features *ft*_*s*_ of an specific image, i.e., *ft*_*s*_ = *f*(*x*; θ_*Smtc*_).

It is worth noting that θ_*Smtc*_ is fixed during the training stage since the proposed DNN framework will be fine-tuned in IQA datasets in which the semantic label is unavailable.

#### 2.2.2. Perceptual-Quality Oriented Feature Extractor

The architecture of perceptual-quality oriented feature extractor *f*(·;θ_*Qlty*_) is quite similar with semantic oriented feature extractor. However, the parameters θ_*Qlty*_ in *f*(·;θ_*Qlty*_) are learnable and independent with θ_*Smtc*_.

The quality-aware features *ft*_*q*_ = *f*(*x*; θ_*Qlty*_) are further leveraged to aggregate the prediction of subjective quality score, i.e., $\hat{q}=h\left(ft_{q};\delta \right)$, in which *q* denotes the subjective quality score (MOS), $\hat{q}$ is the prediction of *q*, and *h*(·;δ) stands for the MOS prediction network given quality-aware features with learnable parameters δ.

#### 2.2.3. Cross-Domain Feature Similarity Predictor

As illustrated in section 1, the cross-domain feature similarity would contribute to the prediction of perceptual quality. However, directly evaluating the similarity between *ft*_*s*_ and *ft*_*q*_ via Minkowski-Distance or Wang-Bovik metric (Wang et al., 2004) is not as efficient, as shown in **Figure 6**. We think the invalidation of the Wang-Bovik metric is mainly attributed to its pixel-wise sensitivity, i.e., any turbulence during the parameter initializing and updating of the DNN framework would result in a significant difference between *ft*_*s*_ and *ft*_*q*_.

To this end, this work proposes to depict the cross-domain feature similarity through a global perspective. Specifically, the similarity is derived from the weighting matrix *W* which is employed to reconstruct *ft*_*q*_ given *ft*_*s*_ via linear regression. Since the *W* is derived from the features amongst all channels, it is less likely to suffer from the instability of the DNN during initializing and updating. The experiments reported in section 3.3 also demonstrate the superiority of our proposed similarity measurement for cross-domain features. In our CDFS-guided BIQA framework, the CDFS is incorporated as follows:

Linear regression is employed for the reconstruction and the weighting matrix *W* could be obtained according to equation 1 and ***Step 1*** in section 2.1

A stack of convolutional layers (denoted as g(·;γ)) is followed to learn the cross-domain feature similarity given *W*.

During the training stage, the cross-domain similarity is employed as a regularization item to supervise the quality prediction network.

#### 2.2.4. Loss Function

The loss function *L* of our proposed network is designed as


(2) $$\begin{array}{r} L_{1}=argmin_{\left[\theta_{Qlty},\delta \right]}\|q-h\left(f\left(x;\theta_{Qlty}\right);\delta \right)\| \end{array}$$

(3) $$\begin{array}{r} L_{2}=argmin_{\left[\theta_{Qlty},\gamma \right]}\|q-g\left(W;\gamma \right)\| \end{array}$$

and


(4) $$\begin{array}{r} L=L_{1}+\lambda L_{2} \end{array}$$

where ‖·‖ denotes the *L*1 norm operator, *W* is calculated according to equation 1, and λ is a hyper parameter controlling the weights of *L*_1_ and *L*_2_.

### 2.3. Implementation Details

We use ResNet50 (He et al., 2016) as the backbone model for both the semantically oriented feature extractor and the perceptual-quality oriented feature extractor. As aforementioned, the pre-trained model on ImageNet (Deng et al., 2009) is used for network initialization. During the training stage, the θ_*Smtc*_ is fixed whilst θ_*Qlty*_ is learnable. In our network, the last two layers of the origin ResNet50, i.e., an average pooling layer and a fully connected layer, are removed to output features *ft*_*s*_ and *ft*_*q*_.

For quality regression, a global average pooling (GAP) layer is used to pool the features *ft*_*q*_ into one-dimensional vectors, then three fully -connected (FC) layers are followed with size 2048-1024-512-1 and activated by ReLu, except for the last layer (activated by sigmoid).

The *g*(·;γ) in cross-domain feature similarity predictor is implemented by 3 three stacked convolutional layers, a GAP layer, and three FC layers. The architectures of convolutional layers are *in*(1) − *out*(32) − *k*(1) − *p*(0), *in*(32) − *out*(64) − *k*(3) − *p*(1), and *in*(64) − *out*(128) − *k*(3) − *p*(1), respectively, where *in*(α) − *out*(β) − *k*(*x*) − *p*(*y*) denotes the input channel size and output channel size is α and β, the kernel size is *x*, and the padding size is *y*. Each of the convolutional layers is followed by a batch normalization layer and a ReLu layer. The GAP layer and the FC layers are the same with quality regression except that the size of FC layers is 128-512-512-1.

The experiment is conducted on Tesla V100P GPUs, while the DNN modules are implemented by Pytorch. The size of minibatch is 24. Adam (Kingma and Ba, 2014) is adopted to optimize the loss function with weight decay 5 × 10^−4^ and learning rate 1 × 10^−5^ for parameters in baseline (ResNet) and 1 × 10^−4^ for other learnable parameters. As mentioned, the parameters in semantic oriented feature extractor is are fixed, i.e., the learning rate is 0 for θ_*Smtc*_.

## 3. Experimental Results

### 3.1. Datasets and Evaluation Metrics

Three image databases including KonIQ-10k (Hosu et al., 2020), LIVE Challenges (LIVEC) (Ghadiyaram and Bovik, 2015), and TID2013 (Ponomarenko et al., 2015) are employed to validate the performance of our proposed network. The KonIQ-10k and LIVEC are authentically distorted image databases containing 10,073 and 1,162 distorted images, respectively, and the TID2013 is a synthetic image database containing 3,000 distorted images.

Two commonly used criteria, Spearman's rank order correlation coefficient (SRCC) and Pearson's linear correlation coefficient (PLCC), are adopted to measure the prediction monotonicity and the prediction accuracy. For each database, 80% images are used for training, and the others are used for testing. The synthetic image database is split according to reference images. All the experiments are under five times random train-test splitting operation, and the median SRCC and PLCC values are reported as final statistics.

### 3.2. Comparison With the State-of-the-Art Methods

Ten BIQA methods are selected for performance comparison, including five hand-crafted based (BRISQUE Mittal et al., 2012, ILNIQE Xu et al., 2016, HOSA Zhang et al., 2015, BPRI Min et al., 2017a, BMPRI Min et al., 2018a) and five DNN-based approaches (SFA Li et al., 2018, DBCNN Zhang et al., 2018b, HyperIQA Su et al., 2020, SDGNet Yang et al., 2019). The experimental results are shown as in Table 1.

**Table 1:** Performance comparison in terms of PCLL and SRCC on KonIQ, LIVEC, and TID2013, respectively.

**SRCC**	**KonIQ**	**LIVEC**	**TID2013**
BRISQUE	0.665	0.608	0.572
ILNIQE	0.507	0.432	0.521
HOSA	0.671	0.640	0.688
BPRI	–	–	0.899
BMPRI	–	–	**0.929**
SFA	0.856	0.812	–
DBCNN	0.875	0.851	–
HyperIQA	0.906	0.859	–
SGDNet	0.903	0.851	0.843
DeepFL	0.877	0.734	0.858
ours	**0.918**	**0.865**	0.899
**PLCC**	**KonIQ**	**LIVEC**	**TID2013**
BRISQUE	0.681	0.645	0.651
ILNIQE	0.523	0.508	0.648
HOSA	0.694	0.678	0.764
BPRI	–	–	0.892
BMPRI	–	–	**0.947**
SFA	0.872	0.833	–
DBCNN	0.884	0.869	–
HyperIQA	0.917	**0.882**	–
SGDNet	0.920	0.872	0.861
DeepFL	0.887	0.769	0.876
ours	**0.928**	0.875	0.880

*Values in bold represents the highest value*.

As shown in Table 1, our method outperforms all the SOTA methods on the two authentic image databases in terms of SRCC. As for PLCC measurement, our method achieves the best performance on KonIQ and competing (the second) performance on LIVEC. This suggests that calculating cross-domain feature similarity for quality prediction refinement is effective. Though we do not especially modify the networks for synthetic image feature extraction, the proposed network has achieved competing performance in TID2013. Specifically, the proposed approach achieves the second-highest performance in terms of SRCC and the third-highest performance in terms of PLCC on TID2013.

### 3.3. Cross-Domain Feature Similarity Visualization

**Figure 5:**
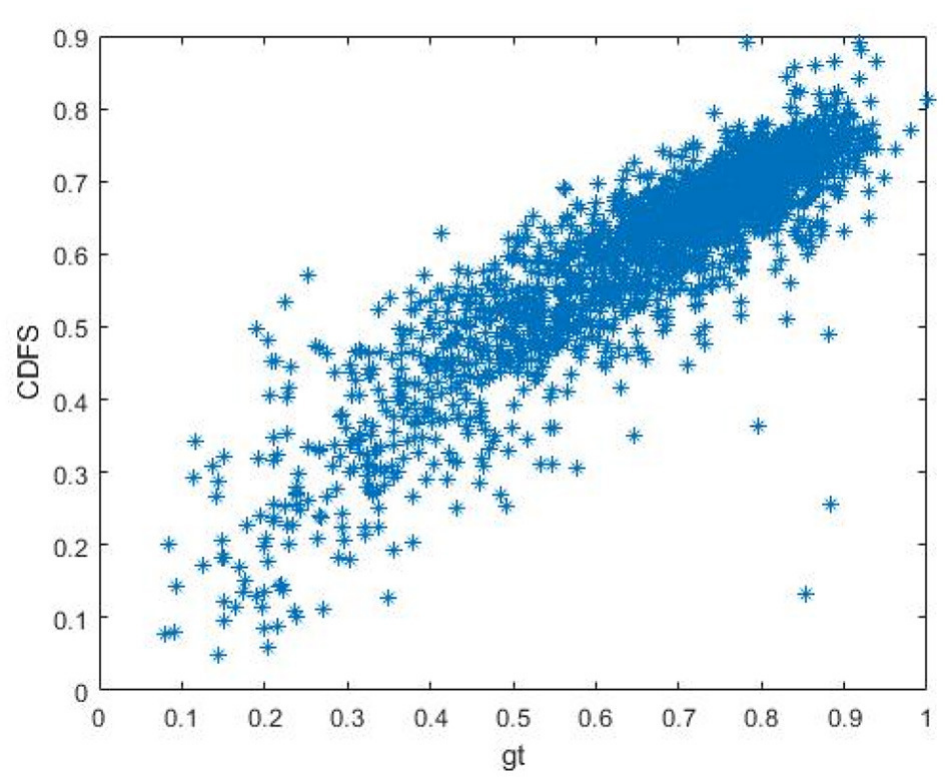
The scatter plot of CDFS vs. MOS on KonIQ.

In order to further illustrate the superiority of our proposed CDFS, we firstly present the scatter plot of CDFS vs. MOS on KonIQ in Figure 5, indicating the CDFS is well correlated with perceptual quality.

In addition, we also investigate several non-learnable approaches for calculating CDFS: (1) $Sim_{1}=mean\left(\frac{2\times ft_{s}\times ft_{q}+C}{ft_{s}^{2}+ft_{q}^{2}+C}\right)$, where *C* denotes the constant to avoid numerical singularity; and (2) *Sim*_2_ = *std*(*eig*(*W*)); (3) $Sim_{3}=mean\left(\frac{2\times \vec{v}\times \vec{1}+C}{{\vec{v}}^{2}+{\vec{1}}^{2}+C}\right)$, and $\vec{v}=eig\left(W\right)$, in which $\vec{1}$ denotes the vectors with the same size as $\vec{v}$ whilst whose elements are all 1.

**Figure 6:**
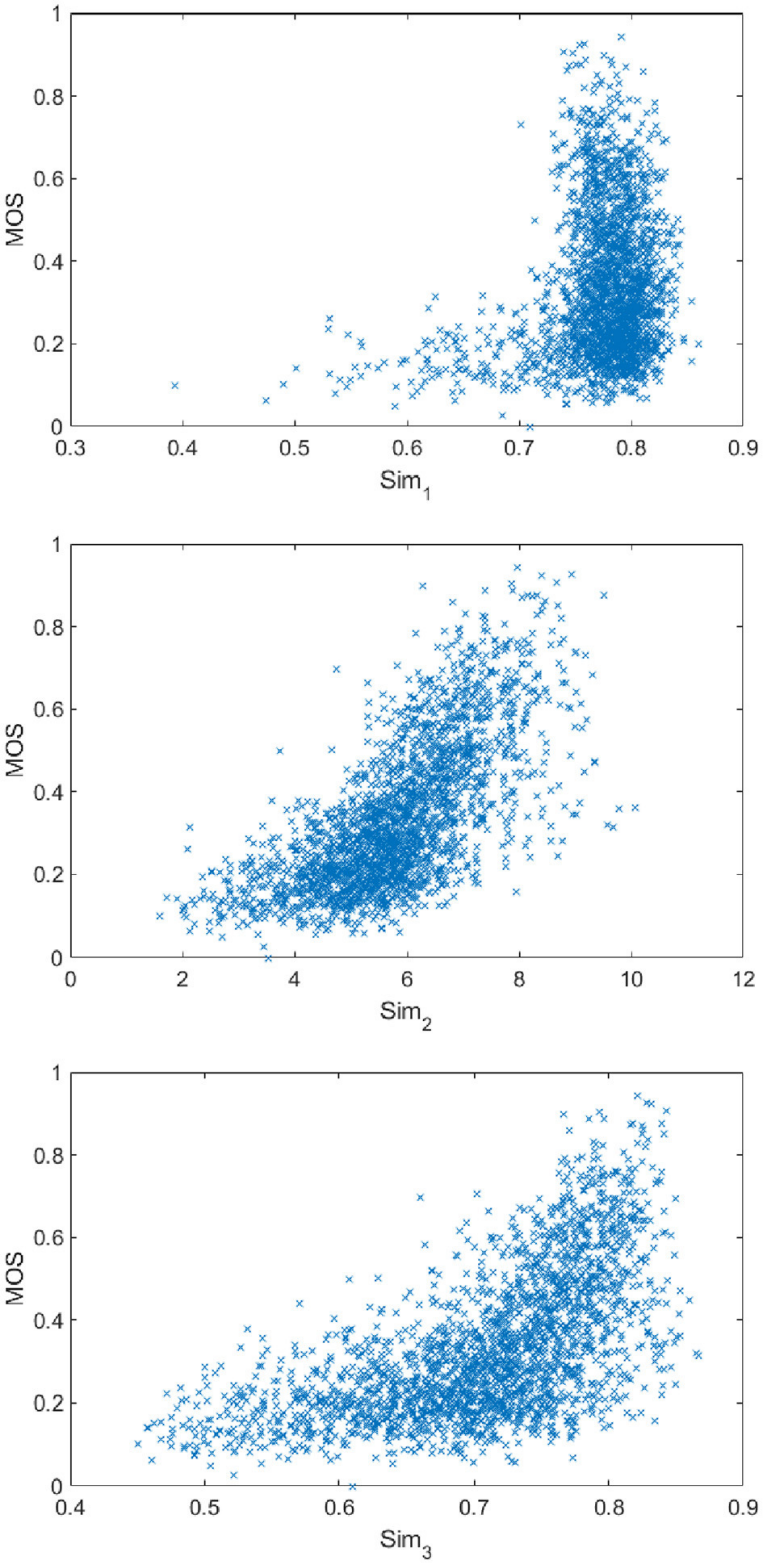
The scatter plot of *Sim*_1_, *Sim*_2_, and *Sim*_3_ vs. MOS on KonIQ.

Therefore, the calculation of *Sim*_1_ is directly comparing the difference between *ft*_*s*_ and *ft*_*q*_, and the calculation of *Sim*_2_ and *Sim*_3_ is based on the *W* derived according to equation 1. As shown in Figure 6, *Sim*_2_ and *Sim*_3_ is more correlated with the subjective score, demonstrating that measuring the cross-domain feature similarity based on *W* is more effective.

### 3.4. Ablation Study

Ablation study is conducted on KonIQ-10k to validate the efficiency of our proposed components, including the ResNet50 backbone (BaseLine), the similarity predictor (SP) obtained by Wang-Bovik metric (SP_wang, similar as *Sim*_1_ in section 3.3), and the similarity predictor derived from the weighting metric *W* (SP_*W*). The results are shown in Table 2, indicating that incorporating a cross-domain similarity predictor could significantly improve the accuracy of quality prediction. Our proposed similarity measurement has achieved a great PLCC improvement (1.8%) compared to SP_wang and a more significant SRCC improvement (2.7%).

**Table 2:** Ablation results in terms of SRCC and PLCC on KonIQ.

**Modules**	**BaseLine**	**+SP**_**wang**	**+SP**_**W**
SRCC	0.842	0.895	0.918
Gain(%)	–	6.3	9.0
PLCC	0.849	0.913	0.928
Gain(%)	–	7.5	9.3

**Figure 7:**
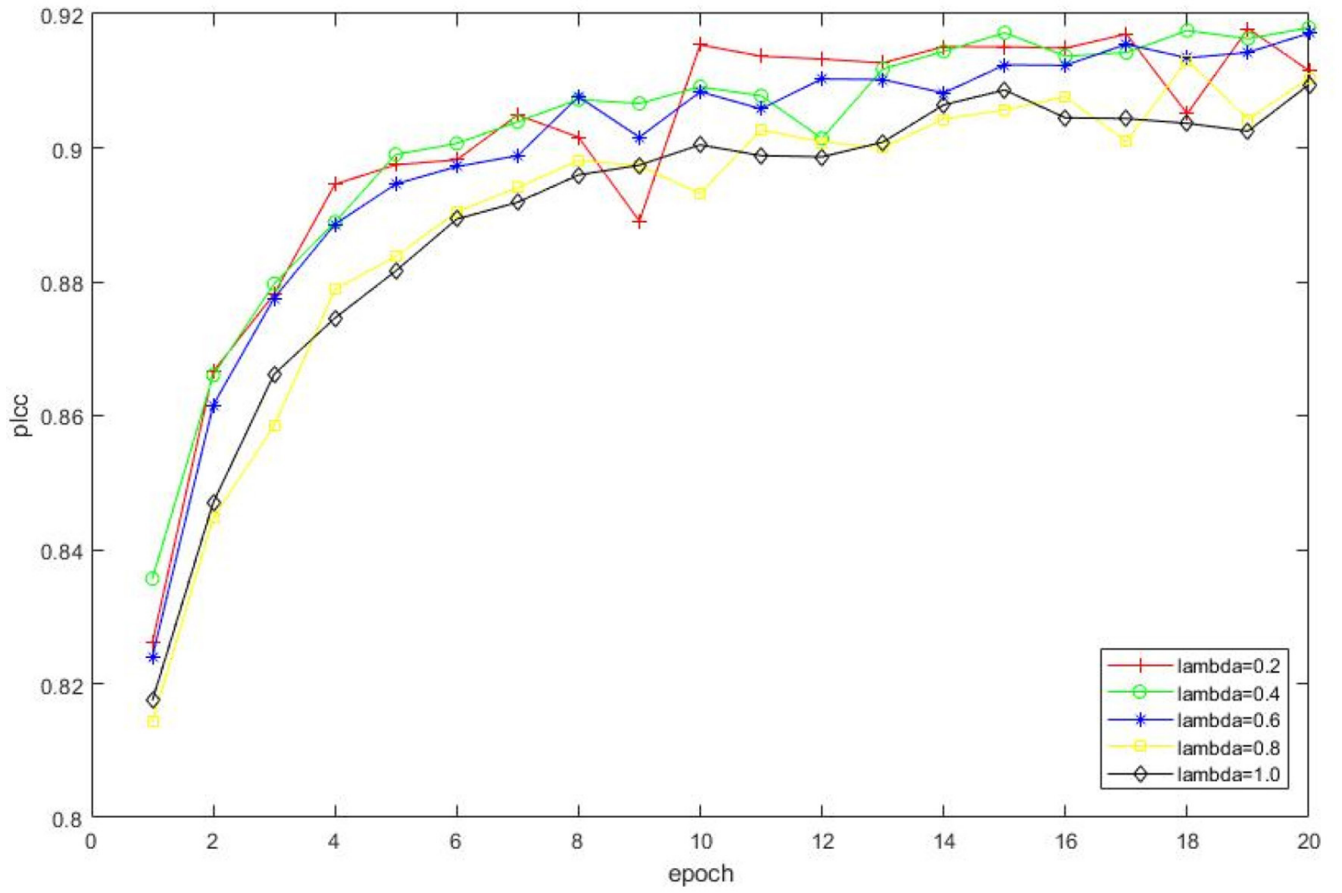
Impact on selections of different λ. The experimental result is conducted on KonIQ, and a total of 20 epochs are involved.

The impact of λ in equation 4 is also investigated, i.e., we set λ = [0.2, 0.4, 0.6, 0.8, 1.0], respectively and observe the corresponding performance as shown in Figure 7. Therefore, we select λ = 0.4 for performance comparison and the following experiments.

### 3.5. Cross-Database Validation

In order to test the generalization ability of our network, we train the model on the entire KonIQ-10k and test on the entire LIVEC. The four most competing IQA models in terms of generalization ability are involved in the comparison, which are PQR (Zeng et al., 2017), DBCNN, HyperIQA, and DeepFL. The validation results are shown in Table 3, indicating the generalization ability of our approach is higher than existing SOTA methods for assessing authentically distorted images.

**Table 3:** Cross data base validation (Trained on KonIQ-10k and Tested on LIVEC).

**Modules**	**DeepFL**	**DBCNN**	**HyperIQA**	**PQR**	**Ours**
SRCC	0.704	0.755	0.770	0.785	0.817
Gain(%)	–	7.2	9.4	11.5	16.1

However, if the network is trained on KonIQ-10k and directly applied for a synthetic image database, its generalization ability is not satisfactory, and the SRCC on TID2013 is only 0.577. That is mainly because the distortion mechanisms between synthetic and authentically distorted image databases are widely different. Training the network solely on authentically -distorted image databases could not learn the specific synthetic distortion patterns such as JPEG compression, transmission errors, or degradation caused by denoising, *etc*.

### 3.6. Further Validation on Other Specific IQA Tasks

In order to further validate the robustness of our BIQA framework toward other specific IQA tasks, the performance of CDFS guided BIQA network is evaluated on CCT (Min et al., 2017b), DHQ (Min et al., 2018b), and SHRQ (Min et al., 2019). The CCT contains 1,320 distorted images with various types of images including natural scene images (NSI), computer graphic images (CGI), and screen content images (SCI); The DHQ contains 1,750 dehazed images generated from 250 real hazy images.; The SHRQ database consists of two subsets, namely: regular and aerial image subsets, which include 360 and 240 dehazed images created from 45 and 30 synthetic hazy images using 8 eight image dehazing algorithms, respectively.

The training pipeline is similar with section 3.1, i.e., 80% of the CCT, DHQ, or SHRQ are involved as the training set and the other 20% is the testing set. Considering that the scale of the subset is not adequate for the training of DNN, we merge the subsets in each datasets. For example, the NSI, CGI, and SCI are merged as the training set of CCT.

As shown in Table 4, the predictions of our CDFS guided BIQA framework shows significant consistency with subjective scores, indicating that our proposed BIQA approach is feasible to be generalized into other types of IQA tasks.

**Table 4:** SRCC and PLCC performance on CCT, DHQ, and SHRQ.

		**SRCC**	**PLCC**
CCT	20-%Test	0.9655	0.9672
	100-%Test	0.5758	0.6193
DHQ	20-%Test	0.9533	0.9223
	100-%Test	0.6819	0.6678
SHRQ	20-%Test	0.8875	0.9082
	100-%Test	0.4233	0.4761

Furthermore, if the network is trained on KonIQ-10k and directly applied on CCT, DHQ, and SHRQ, the accuracy is not satisfactory, as shown in Table 4. Such phenomenon is similar to the cross-database validation results discussed in section 3.5, indicating that training the network solely on authentically-distorted natural image databases could not sufficiently learn the quality-aware features for CGI, SCI, etc.

## 4. Conclusion

This work aims to evaluate the perceptual quality based on cross-domain feature similarity. The experimental results on KonIQ, LIVEC, and TID2013 demonstrate the superiority of our proposed methods.

We would further investigate such CDFS-incorporated BIQA framework in the following aspects: (1) investigating more efficient approaches of CDFS measurement; (2) investigating more types of DNN baselines in addition to ResNet.

## Data Availability Statement

The original contributions presented in the study are included in the article/supplementary material, further inquiries can be directed to the corresponding author.

## Author Contributions

CF established the BIQA framework and adjusted the architecture for better performance. LY and CF conducted the experiments and wrote the manuscripts. QZ designed the original method, and provided resource support (e.g., GPUs) for this manuscript. All authors contributed to the article and approved the submitted version.

## Funding

This work is supported by the National Key R&D Program of China under Grant No. 2021YFF0900503, the National Natural Science Foundation of China under Grant Nos. 61971383 and 61631016, and the Fundamental Research Funds for the Central Universities.

## Conflict of Interest

The authors declare that the research was conducted in the absence of any commercial or financial relationships that could be construed as a potential conflict of interest.

## Publisher's Note

All claims expressed in this article are solely those of the authors and do not necessarily represent those of their affiliated organizations, or those of the publisher, the editors and the reviewers. Any product that may be evaluated in this article, or claim that may be made by its manufacturer, is not guaranteed or endorsed by the publisher.
